# Epidemiological study of HPV infection in 40,693 women in Putian: a population study based on screening for high-risk HPV infection

**DOI:** 10.1186/s12879-022-07893-3

**Published:** 2022-11-28

**Authors:** Zhanfei Chen, Hua Lin, Jinyuan Zheng, Lili Cai, Zhonghui Chen, Jinqiu Li, Liumin Yu

**Affiliations:** 1 The Affiliated Hospital of Putian University, Putian University, No.999 Dongzhen East Road, Licheng District, Putian, Fujian China; 2 Key Laboratory of Medical Microecology, Putian University, Fujian Province University, No.1133 Xueyuan Middle Street, Chengxiang District, Putian, Fujian China; 3 Center Blood Station, No.138 Guanglin Street, Dongzhen West Road, Chengxiang District, Putian, Fujian China

**Keywords:** Putian City, Fujian Province, Human papillomavirus, High-risk genotypes, Epidemiology, Vaccine

## Abstract

**Background:**

The infection rate of human papillomavirus (HPV) is high in the coastal regions of China. However, the infection rate among high-risk genotypes of women in Putian City is unknown. Therefore, this study aimed to analyse the epidemiology of high-risk HPV infection among women in Putian and provide a reference for the diagnosis, treatment and vaccination of cervical cancer in this region.

**Methods:**

The data used were obtained from the Chinese government’s public health program (“Cervical and Breast Cancer Screening Project”). A total of 40,693 female cervical cell exfoliation samples screened for high-risk HPV at the Affiliated Hospital of Putian University from July 2020 to December 2021 were enrolled. DNA was extracted using a fully automatic extractor. Then, 14 high-risk genotypes of HPV were detected by polymerase chain reaction. The characteristics of HPV infection, distribution of high-risk genotypes, infection types and thinprep cytologic test (TCT) classification at different age groups were analysed.

**Results:**

Among the 40,693 samples, 3899 were infected with HPV, with an infection rate of 9.6%. Accordingly, HPV infection rates gradually increased with age, and statistically significant differences were observed among age groups (*χ*^*2*^ = 74.03, *P* < 0.01). The infection rates of high-risk HPV52, HPV58 and HPV16 were in the top three and increased with age. Single infection was dominant (84.7%), followed by double infections (12.7%). The cervical cytology of 3899 HPV-positive people can be classified into negative for intraepithelial lesion and malignancy (NILM, 88.0%), atypical squamous cells of undetermined significance (ASC-US, 6.6%), atypical squamous cells—cannot exclude high-grade squamous intraepithelial lesion (ASC-H, 1.4%), low-grade squamous intraepithelial lesion (LSIL, 3.2%) and high-grade squamous intraepithelial lesion (HSIL, 0.8%). HPV16 infection rate increased with increasing severity of cervical cytology (*χ*^*2*^_*trend*_ = 43.64, *P* < 0.01), whereas the infection rates of HPV52 (*χ*^*2*^_*trend*_ = 13.89, *P* < 0.01) and HPV58 (*χ*^*2*^_*trend*_ = 13.50, *P* < 0.01) showed opposite trends.

**Conclusion:**

The infection rate of female HPV high-risk screening in this region was 9.6% and mainly involved single infections. In addition, HPV16, HPV52 and HPV58 were closely related to the severity of cervical cytology. Effective screening, vaccination and education are needed. The 9-valent vaccine will be effective in reducing cervical pre-invasive disease. It would also be reasonable to state that the rising trend in HPV infection and high grade cytology with age emphasises the need to target older women with screening. Vaccination of younger women (aged ≤ 25) will lay the foundation for better cancer outcomes in the future.

## Introduction

Cervical cancer ranks fourth among malignant tumours in women worldwide. The prevalence rate is increasing annually and has affected young people. In 2018, the World Health Organization (WHO) launched a call to accelerate the elimination of cervical cancer as a global public health problem. In recent years, regional differences have been observed in cervical cancer screening. Specifically, the screening coverage, testing methods and post-screening management differ among high-, middle- and low-income countries. This situation reflects complex factors related to the economic strength of the government, organised population-based programs, monitoring and quality assurance measures and female participation rates [1–3]. In 2009, China launched a national public health program to curb cervical cancer [4]. In comparison with high-income countries, cervical cancer prevention in China remains unsatisfactory. Adequate diagnosis, follow-up and management of positive results are needed for a more effective screening [2]. The Chinese government’s current efforts to implement an organised population-based screening program and reasonable post-screening management will lead to more positive results [5, 6].

Human papillomavirus (HPV) is a double-stranded DNA virus (without an envelope) of the papillomavirusidae [7]. It is highly contagious and can infect the human epidermis and mucous membrane, leading to cervical intraepithelial lesions and cervical cancer [8]. In terms of pathogenicity, HPV viruses can be classified as high- or low-risk types. The high-risk type is closely related to various cervical intraepithelial neoplasias, cervical cancers and other malignant lesions [9]. By contrast, the low-risk type is mainly involved in multiple benign lesions of the external genital tract [10].

Cervical cancer screening methods include conventional cytology, liquid-based cytology, visual inspection with acetic acid, nucleic acid testing and cytology based on Romanovsky-Giemsa staining. However, HPV nucleic acid testing, especially high-risk HPV, has shown greater advantages in improving the detection rate of cervical intraepithelial neoplasia (CIN) 2+ and reducing the incidence of cervical cancer [11–13]. High-risk HPV is closely related to cervical cancer. Approximately 70% of cervical cancer and pre-cancerous lesions are associated with HPV16 and HPV18 [14]. However, the predominant HPV genotypes may result in different outcomes at different stages of the disease [15, 16]. HPV52 and HPV16 are dominant in CIN1, while HPV16 and HPV58 are found in CIN2/3 [17]. Cervical cancer has been linked to persistent infection with high-risk HPV [18, 19]. Therefore, regular HPV detection is a critical measure to prevent and treat HPV-related diseases. If HPV16 or HPV18 infection is detected, colposcopy is also required to obtain accurate diagnostic information [20, 21].

The epidemiological characteristics of cervical cancer vary remarkably across different geographical locations. Approximately 90% of the world’s cervical cancer occurs in countries without effective HPV screening and vaccination [22]. However, formal screening programs have benefited many countries, and cancer prevalence and mortality have decreased by more than half in the past 30 years [22, 23]. Therefore, organised cervical cytology, pre-invasive disease treatment and vaccination are key measures to reduce the incidence of cervical cancer. High-priced HPV vaccines can protect against more HPV subtypes. However, due to limitations such as price and quantity, the optimal vaccination time should not be missed by blindly pursuing high-priced vaccines. Vaccine distribution should be effectively and rationally distributed according to HPV epidemiological characteristics in different regions. Information from (regular, large) HPV screening is needed to corroborate with the epidemiological results. However, limited studies have reported the characteristics of HPV infection in Putian City, Fujian Province in the past decade. Investigating the epidemiology of HPV in this region is of great help in choosing vaccines effectively. In this study, the results of large-scale female HPV high-risk infection screening in Putian City in the past 2 years were collected to explore the status of high-risk HPV infection and the distribution of genotypes in this region. Ultimately, this study aimed to provide a practical reference for the epidemiology, diagnosis, treatment and vaccination of cervical cancer in this region.

## Materials and methods

### Study population

The data for this study were obtained from the Chinese government’s public health program (“Cervical and Breast Cancer Screening Project”), under which regular tests are conducted every year. The present survey was conducted in Putian City, Fujian province. A total of 40,693 females who were screened for high-risk HPV at the Affiliated Hospital of Putian University from July 2020 to December 2021 were enrolled. The age range was 30–70 years (50.1 ± 7.9). The inclusion criteria are as follows: no history of hysterectomy or cervical surgery, no severe endocrine or autoimmune diseases, abstinence before the examination, no vaginal administration and non-pregnancy. The exclusion criteria are as follows: incomplete information, history of cervical conization and hysterectomy, history of cervical treatment in the last 3 months, acute reproductive tract inflammation, elimination of secondary re-examination data and exclusion of common gynaecological diseases (e.g., cervical cancer, ovarian cancer and uterine leiomyoma). All tests were performed with the informed consent of the enrolled women. This study was approved by the Ethics Committee of the Affiliated Hospital of Putian University (202224).

### Specimen collection and HPV genotyping

Cervical samples were obtained from women by using a cytobrush according to the instructions, and the samples were used for genomic DNA extraction. The samples of cervical exfoliated cells were thoroughly vortexed and mixed. Then, 350 µl of liquid was absorbed into the nucleic acid extraction reagent. HPV DNA was extracted using a nucleic acid extractor. DNA (5 µl) was mixed well with amplification reagent (20 µl). After capping the amplification tube and quick centrifugation, the PCR amplification reaction was carried out via real-time fluorescence quantitative PCR. All procedures were performed strictly according to the kit’s instructions.

### ThinPrep cytologic test (TCT)

Specimens that were positive for HPV genotyping were directly used for TCT. Two experienced cytopathologists independently diagnosed the liquid cytology specimens. A third cell pathologist reviewed the sample when the diagnosis of the two cytopathologists differed. According to Bethesda’s system, cytological results were classified into negative for intraepithelial lesion and malignancy (NILM), atypical squamous cells of undetermined significance (ASC-US), atypical squamous cells—cannot exclude high-grade squamous intraepithelial lesion (ASC-H), low-grade squamous intraepithelial lesion (LSIL) and high-grade squamous intraepithelial lesion (HSIL) [24].

### Statistical analysis

Data were analysed using GraphPad Prism 7.0 software. An interval of 95% confidence (95% CI) was calculated for HPV prevalence. The *χ*^*2*^ test was applied to compare the infection rate of different age groups and the infection rate of high-risk genotypes according to additional TCT classifications. Differences were considered statistically significant if the difference was *P* < 0.05.

## Results

### Characteristics of HPV infection in general and specific age groups

In this study, 40,693 cases were analysed. A total of 3899 cases were HPV positive, and the overall HPV infection rate was 9.6% (95% confidence interval [CI] 9.3–9.9%, Table 1). According to the age group, they were divided into four groups, namely, 30–39, 40–49, 50–59 and 60–70 years, and the majority of people were aged 50–59 and 40–49 years. Notably, the HPV infection rate in the four groups increased gradually with increasing age, and the lowest value was recorded at the age of 30–39 (8.2%, 95% CI 7.5–9.0%), while the highest value was recorded at the age of 60–70 (12.3%, 95% CI 11.4–13.2%). The infection rates of the 50–59 year age group (10.0%, 95% CI 9.5–10.4%) and 60–70 year age group (12.3%, 95% CI 11.4–13.2%) were higher than the overall infection rate (9.6%, 95% CI 9.3–9.9%). The difference in HPV infection rates among different age groups was statistically significant (*χ*^*2*^ = 74.03, *P* < 0.01; Table 1).

**Table 1:** Distribution of HPV infection among age groups in 40,693 women

Age group (y)	Sample size	Positive no.	Prevalence (%)	95% CI	*χ*^*2*^ (*P*)
30–39	5219	429	8.2	7.5–9.0	74.03 (< 0.01)
40–49	13,370	1147	8.6	8.1–9.1	
50–59	16,940	1687	10.0	9.5–10.4	
60–70	5164	636	12.3	11.4–13.2	
Total no.	40,693	3899	9.6	9.3–9.9	

**Fig. 1:**
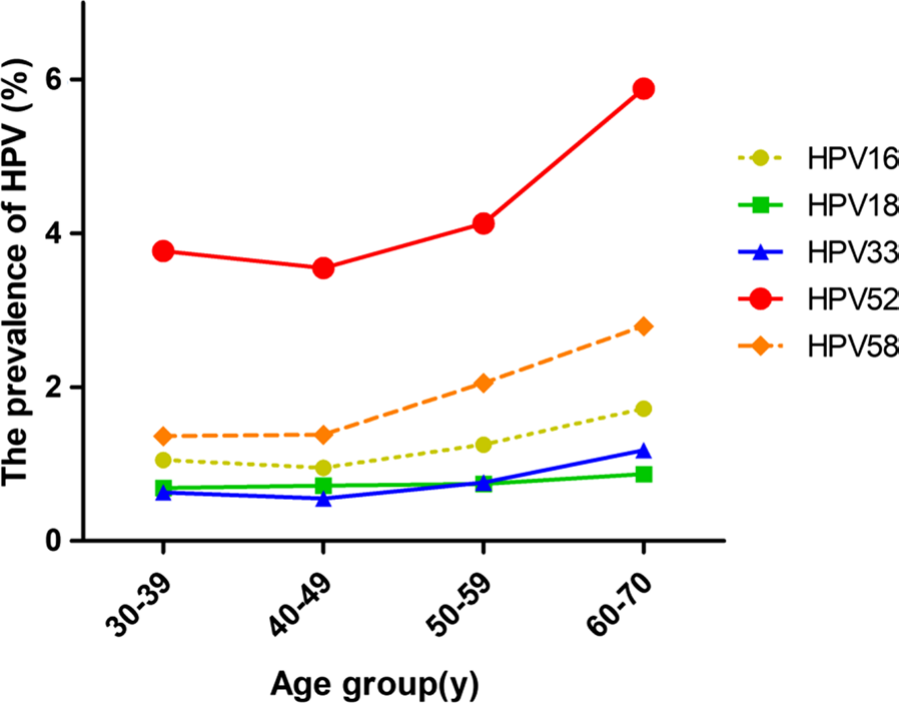
The HPV prevalence distribution in age groups for the five highest-risk types

This study summarised the infection situation of 14 high-risk genotypes of HPV in four age groups (Table 2). The infection rates of HPV16, HPV18, HPV33, HPV52 and HPV58 were in the top five in the total and different groups. These groups include HPV52 (4.1%, 95% CI 3.9–4.3%), HPV58 (1.8%, 95% CI 1.7–2.0%), HPV16 (1.2%, 95% CI 1.1–1.3%), HPV18 (0.7%, 95% CI 0.7–0.8%) and HPV33 (0.7%, 95% CI 0.6–0.8%). Moreover, the infection rates of the first three HPV genotypes increased with increasing age (Fig. 1). The five high-risk types with the lowest overall infection rates were HPV68 (0.3%, 95% CI 0.2–0.3%), HPV66 (0.3%, 95% CI 0.2–0.3%), HPV31 (0.2%, 95% CI 0.1–0.2%), HPV35 (0.2%, 95% CI 0.1–0.2%) and HPV45 (0.1%, 95 CI 0.1–0.1%). This finding indicates the characteristics of the infection rate of the high-risk type of HPV in this region.

**Table 2:** Distribution of 14 high-risk HPV types in age groups

HPV type	30–39 y (n = 5219)		40–49 y (n = 13,370)		50–59 y (n = 16,940)		60–70 y (n = 5164)		All ages (n = 40,693)	
	Positive no. (%)	95% CI	Positive no. (%)	95% CI	Positive no. (%)	95% CI	Positive no. (%)	95% CI	Positive no. (%)	95% CI
16	55 (1.1)	0.8–1.3	127 (1.0)	0.8–1.1	212 (1.3)	1.1–1.4	89 (1.7)	1.4–2.1	483 (1.2)	1.1–1.3
18	36 (0.7)	0.5–0.9	96 (0.7)	0.6–0.9	125 (0.7)	0.6–0.9	45 (0.9)	0.6–1.1	302 (0.7)	0.7–0.8
31	6 (0.1)	0.0**–**0.2	24 (0.2)	0.1**–**0.3	36 (0.2)	0.1**–**0.3	7 (0.1)	0.0**–**0.2	73 (0.2)	0.1**–**0.2
33	33 (0.6)	0.4–0.8	73 (0.5)	0.4–0.7	128 (0.8)	0.6–0.9	61 (1.2)	0.9–1.5	295 (0.7)	0.6–0.8
35	10 (0.2)	0.1**–**0.3	15 (0.1)	0.1**–**0.2	34 (0.2)	0.1**–**0.3	6 (0.1)	0.0**–**0.2	65 (0.2)	0.1**–**0.2
39	25 (0.5)	0.3**–**0.7	78 (0.6)	0.5**–**0.7	84 (0.5)	0.4**–**0.6	24 (0.5)	0.3**–**0.6	211 (0.5)	0.4**–**0.6
45	4 (0.1)	0.0**–**0.2	4 (0.0)	0.0**–**0.1	23 (0.1)	0.1**–**0.2	4 (0.1)	0.0**–**0.2	35 (0.1)	0.1**–**0.1
51	20 (0.4)	0.2**–**0.6	63 (0.5)	0.4**–**0.6	74 (0.4)	0.3**–**0.5	26 (0.5)	0.3**–**0.7	183 (0.4)	0.4**–**0.5
52	197 (3.8)	3.3–4.3	475 (3.6)	3.2–3.9	699 (4.1)	3.8–4.4	303 (5.9)	5.2–6.5	1674 (4.1)	3.9–4.3
56	13 (0.2)	0.1–0.4	44 (0.3)	0.2–0.4	108 (0.6)	0.5–0.8	39 (0.8)	0.5–1.0	204 (0.5)	0.4–0.6
58	71 (1.4)	1.0–1.7	185 (1.4)	1.2–1.6	348 (2.1)	1.8–2.3	144 (2.8)	2.3–3.2	748 (1.8)	1.7–2.0
59	14 (0.3)	0.1–0.4	36 (0.3)	0.2–0.4	46 (0.3)	0.2–0.3	27 (0.5)	0.3–0.7	123 (0.3)	0.2–0.4
66	6 (0.1)	0.0–0.2	40 (0.3)	0.2–0.4	47 (0.3)	0.2–0.4	16 (0.3)	0.2–0.5	109 (0.3)	0.2–0.3
68	7 (0.1)	0.0–0.2	33 (0.2)	0.2–0.3	45 (0.3)	0.2–0.3	27 (0.5)	0.3–0.7	112 (0.3)	0.2–0.3

### Distribution of HPV infection types in age groups and high-risk genotypes

The four types of HPV infections included single, double, triple and ≥ quadruple infection. The results showed (Table 3) that single infection accounted for the highest proportion among the four age groups, and the overall single infection rate reached 84.7% (95% CI 83.6–85.9%). This group was followed by double infections. The overall infection rate was 12.7% (95% CI 11.7–13.7%). Interestingly, the single infection rate was the highest in the 40–49 year age group (88.5%, 95% CI 86.6–90.3%), and the multiple infection rates were the lowest (double 10.5%, triple 1.0%, ≥ quadruple 0.1%). However, participants aged 60–70 years showed the opposite trend, in which the single infection rate was the lowest (78.6%, 95% CI 75.4–81.8%). Multiple infection rates (double 15.9%, triple 4.4%, ≥ quadruple 1.1%) were the highest, indicating that different mechanisms of HPV infection were involved in these two age groups of women.

**Table 3:** Distribution of different infection categories in age groups

Age group (y)	Positive no	Single infection		Double infections		Triple infections		≥ Quadruple infections	
		Positive no. (%)	95% CI	Positive no. (%)	95% CI	Positive no. (%)	95% CI	Positive no. (%)	95% CI
30–39	429	372 (86.7)	83.5–90.0	48 (11.2)	8.2–14.2	8 (1.9)	0.6–3.1	1 (0.2)	0.0–0.7
40–49	1147	1015 (88.5)	86.6–90.3	120 (10.5)	8.7–12.2	11 (1.0)	0.4–1.5	1 (0.1)	0.0–0.3
50–59	1687	1417 (84.0)	82.2–85.7	226 (13.4)	11.8–15.0	39 (2.3)	1.6–3.0	5 (0.3)	0.0–0.6
60–70	636	500 (78.6)	75.4–81.8	101 (15.9)	13.0–18.7	28 (4.4)	2.8–6.0	7 (1.1)	0.3–1.9
Total no	3899	3304 (84.7)	83.6–85.9	495 (12.7)	11.7–13.7	86 (2.2)	1.7–2.7	14 (0.4)	0.2–0.5

This paper attempted to explore the distribution of infection types of different high-risk genotypes of HPV. The results (Table 4) showed that a single infection type was still dominant, followed by double infection. In single, double, and triple infections, the top three were HPV52, HPV58 and HPV16. In ≥ quadruple infections, the genotypes were HPV52, HPV56 and HPV59. Generally, HPV52 was the primary type of HPV infection.

**Table 4:** Distribution of 14 high-risk HPV types in different infection categories

HPV type	Single infection		Double infections		Triple infections		≥ Quadruple infections	
	Positive no. (%)	95% CI	Positive no. (%)	95% CI	Positive no. (%)	95% CI	Positive no. (%)	95% CI
16	333 (0.8)	0.7–0.9	113 (0.3)	0.2–0.3	35 (0.1)	0.1–0.1	2 (0.0)	0.0–0.0
18	196 (0.5)	0.4–0.5	86 (0.2)	0.2–0.3	17 (0.0)	0.0–0.1	3 (0.0)	0.0–0.0
31	48 (0.1)	0.1–0.2	18 (0.0)	0.0–0.1	6 (0.0)	0.0–0.0	1 (0.0)	0.0–0.0
33	179 (0.4)	0.4–0.5	97 (0.2)	0.2–0.3	16 (0.0)	0.0–0.1	3 (0.0)	0.0–0.0
35	34 (0.1)	0.1–0.1	17 (0.0)	0.0–0.1	9 (0.0)	0.0–0.0	5 (0.0)	0.0–0.0
39	148 (0.4)	0.3–0.4	48 (0.1)	0.1–0.2	11 (0.0)	0.0–0.0	4 (0.0)	0.0–0.0
45	25 (0.1)	0.0–0.1	6 (0.0)	0.0–0.0	2 (0.0)	0.0–0.0	2 (0.0)	0.0–0.0
51	124 (0.3)	0.3–0.4	35 (0.1)	0.1–0.1	20 (0.0)	0.0–0.1	4 (0.0)	0.0–0.0
52	1357 (3.3)	3.2–3.5	252 (0.6)	0.5–0.7	54 (0.1)	0.1–0.2	11 (0.0)	0.0–0.0
56	124 (0.3)	0.3–0.4	47 (0.1)	0.1–0.1	22 (0.1)	0.0–0.1	11 (0.0)	0.0–0.0
58	510 (1.3)	1.1–1.4	190 (0.5)	0.4–0.5	45 (0.1)	0.1–0.1	3 (0.0)	0.0–0.0
59	76 (0.2)	0.1–0.2	30 (0.1)	0.0–0.1	9 (0.0)	0.0–0.0	8 (0.0)	0.0–0.0
66	73 (0.2)	0.1–0.2	23 (0.1)	0.0–0.1	7 (0.0)	0.0–0.0	6 (0.0)	0.0–0.0
68	77 (0.2)	0.1–0.2	28 (0.01)	0.0–0.1	5 (0.0)	0.0–0.0	2 (0.0)	0.0–0.0

### Cervical cytological status of age groups and high-risk genotypes

The cervical cytological status of 3899 HPV-positive people was analysed, mainly involved NILM (88.0%), in which ASC-US, ASC-H, LSIL and HSIL accounted for 6.6%, 1.4%, 3.2% and 0.8%, respectively (Table 5). In NILM, the 40–49 year age group had the highest rate (88%), while the 60–70 year age group had the lowest rate (85.8%). The 60–70 year age group (8.3%) had the highest rate in ASC-US, and the 50–59 year age group accounted for the most in ASC-H. In LSIL and HSIL, the 50–59 and 60–70 year age group showed high proportion (Table 5). The results indicate that after the age of 40, the risk of cervical carcinogenesis in HPV-positive people might gradually increase.

**Table 5:** Distribution of TCT results in HPV-positive specimens by age group

Age group (y)	Positive no	NILM	ASC-US	ASC-H	LSIL	HSIL
		Positive no. (%)	Positive no. (%)	Positive no. (%)	Positive no. (%)	Positive no. (%)
30–39	429	376 (87.6)	30 (7.0)	6 (1.4)	17 (4.0)	0 (0.0)
40–49	1147	1018 (88.8)	71 (6.2)	13 (1.1)	36 (3.1)	9 (0.8)
50–59	1687	1490 (88.3)	105 (6.2)	27 (1.6)	48 (2.8)	17 (1.0)
60–70	636	546 (85.8)	53 (8.3)	9 (1.4)	23 (3.6)	5 (0.8)
Total no	3899	3430 (88.0)	259 (6.6)	55 (1.4)	124 (3.2)	31 (0.8)

**Fig. 2:**
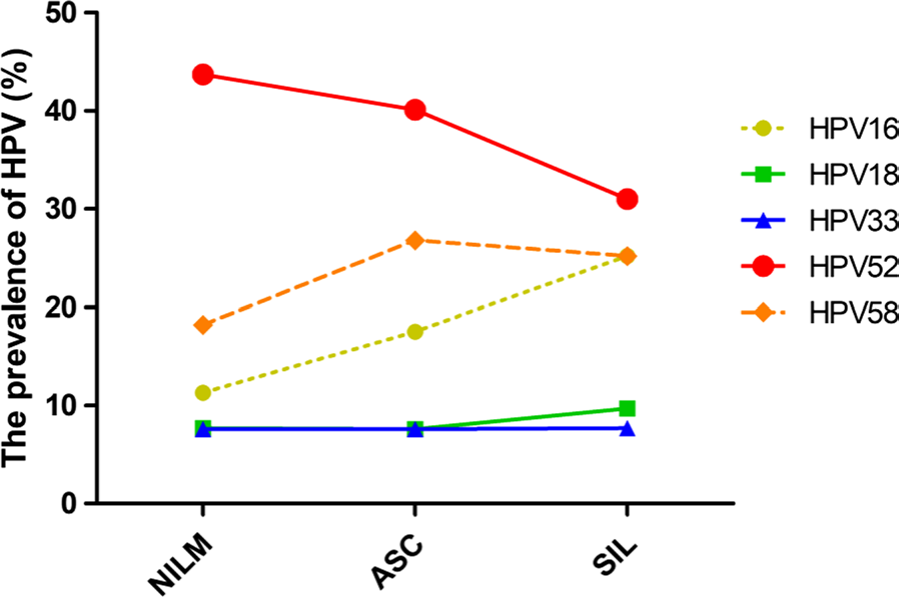
The HPV prevalence distribution in TCT classification for the five highest-risk types

The relationship between high-risk genotypes and TCT results in HPV-positive people was also analysed (Table 6). The top five genotypes in NILM and ASC-US were HPV52, HPV58, HPV16, HPV18 and HPV33. The descending genotypes of ASC-H were HPV58, HPV52, HPV16, HPV33 and HPV18. The descending genotypes of LSIL were HPV52, HPV58, HPV16, HPV18 and HPV39. HSIL included HPV16, HPV58, HPV52, HPV51 and HPV33. In the case of abnormal TCT, the infection rate of HPV16 increased with increasing severity of cervical cytology (*χ*^*2*^_*trend*_ = 43.64, *P* < 0.01), while the infection rates of HPV52 (*χ*^*2*^_*trend*_ = 13.89, *P* < 0.01) and HPV58 (*χ*^*2*^_*trend*_ = 13.50, *P* < 0.01) showed the opposite trend (Fig. 2).

**Table 6:** The relationship between high-risk genotypes and TCT results in HPV-positive specimens

HPV type	NILM (n = 3430)	ASC-US (n = 259)	ASC-H (n = 55)	LSIL (n = 124)	HSIL (n = 31)	*χ*^*2*^ (*P*)	*χ*^*2*^_trend_ (*P*)
	Positive no. (%)	Positive no. (%)	Positive no. (%)	Positive no. (%)	Positive no. (%)		
16	389 (11.3)	43 (16.6)	12 (21.8)	21 (16.9)	18 (58.1)	**74.16 (**< **0.01)**	**43.64 (**< **0.01)**
18	263 (7.7)	19 (7.3)	5 (9.1)	14 (11.3)	1 (3.2)	3.30 (0.51)	0.47 (0.49)
31	59 (1.7)	9 (3.5)	2 (3.6)	3 (2.4)	0 (0.0)	5.78 (0.22)	0.97 (0.33)
33	259 (7.6)	18 (6.9)	6 (10.9)	10 (8.1)	2 (6.5)	1.12 (0.89)	0.05 (0.82)
35	56 (1.6)	4 (1.5)	1 (1.8)	3 (2.4)	1 (3.2)	0.94 (0.92)	0.67 (0.41)
39	184 (5.4)	13 (5.0)	2 (3.6)	11 (8.9)	1 (3.2)	3.62 (0.46)	0.53 (0.47)
45	31 (0.9)	3 (1.2)	0 (0.0)	1 (0.8)	0 (0.0)	0.99 (0.91)	0.18 (0.67)
51	154 (4.5)	15 (5.8)	2 (3.6)	9 (7.3)	3 (9.7)	4.70 (0.32)	3.56 (0.06)
52	1500 (43.7)	109 (42.1)	17 (30.9)	44 (35.5)	4 (12.9)	**18.43 (**< **0.01)**	**13.89 (**< **0.01)**
56	186 (5.4)	9 (3.5)	0 (0.0)	8 (6.5)	1 (3.2)	5.52 (0.24)	0.72 (0.39)
58	625 (18.2)	63 (24.3)	21 (38.2)	32 (25.8)	7 (22.6)	**23.01 (**< **0.01)**	**13.50 (**< **0.01)**
59	107 (3.1)	8 (3.1)	1 (1.8)	7 (5.6)	0 (0.0)	3.87 (0.42)	0.24 (0.63)
66	95 (2.8)	8 (3.1)	1 (1.8)	5 (4.0)	0 (0.0)	1.87 (0.76)	0.01 (0.91)
68	103 (3.0)	7 (2.7)	0 (0.0)	2 (1.6)	0 (0.0)	3.48 (0.48)	2.65 (0.10)

Bold indicates a statistically significant

## Discussion

Malignant tumours of the female reproductive tract are prevalent in developing countries [25]. Since the introduction of cervical cancer screening in China in 2009, the incidence and mortality of cervical cancer in Chinese women have decreased. However, the prevention and treatment of cervical cancer in China has not obtained promising results, and cervical cancer has become prevalent in young people [26, 27]. Globally, young women (< 25 years of age) are prone to peak HPV infection rates, whereas a second peak in older women has been observed in regions such as China, East Africa and Latin America [28–30]. A second peak in invasive cancers used to be seen (in the UK) in the early 1990s, but this has largely disappeared as screening has detected and treated much of the CIN in younger women. There was still a small rise in 60–70 year old women [31]. HPV infection plays an essential role in the development and occurrence of cervical cancer. However, the HPV infection rate varies among different countries and regions [32], which may be related to the geographical location, living environment and living habits of the resident population [33, 34].

This study was a large-scale HPV screening study among women in the region, and the results were closely related to the unique research population. Results showed that the overall HPV infection rate in the regular screening population was 9.6%, which was significantly lower than the HPV infection rate in women who visited the gynaecology department of hospitals [35, 36]. Although the hospital (gynaecology) patients were a selected group and were therefore likely to have a higher overall rate of HPV positive testing, the trends with age were the same in both the general population and the hospital population [33, 37, 38]. These conditions may be related to the lack of detection and treatment of HPV infection and precancer at an earlier age. Associations were observed among HPV infection and education, smoking, social behaviours and rurality [24, 39]. In the present study, the infection rate of high-risk HPV in women was also consistent with this characteristic. The infection rate people aged 50–70 years was higher than the overall infection rate, suggesting that middle-aged and older women had a high risk of HPV infection. The infection rates of high-risk HPV52, HPV58 and HPV16 increased with age, and HPV52 was the genotype with the highest infection rate, consistent with Taiwan and southern China [38, 40] but different from the United States [41]. The infection rate of HPV16 only ranked third in the present study.

Notably, the 60–70 year age group had the highest HPV infection rate, and compared with the previous groups, the single infection rate was the lowest, while the multiple infection rates were the highest. This condition may be related to decreased body immunity and the reactivation of menopausal latent HPV [42, 43]. Multiple infections in 60–70 group might represent more than one episode of infection as well as a multiple infection. Women in this group are likely to show persistent HPV infection [25, 44] and have a high risk of cervical cancer. Therefore, it comprehensive and regular cervical screening should be strengthened for HPV-positive middle-aged and older women [22, 44, 45].

This study also deeply analysed the relationship between cervical cytology and HPV infection. The results showed that with the increase in age group, the risk of malignant transformation of cervical cells (≥ ASC-US) in HPV-positive people gradually increased, thus confirming the high risk of cervical cancer in middle-aged and older women. Interestingly, the proportion of high-risk genotypes differed in different stages of cervical cytological abnormalities. For example, HPV16 ranked first in HSIL and only ranked third in non-HSIL. The HPV52 phenotype was opposite to that of HPV16. HPV16, HPV52 and HPV58 were the top three types of CIN in China [17]. This study suggested that the infection rates of HPV16, HPV52 and HPV58 were closely related to the severity of cervical cytology, which was consistent with previous views [17]. The results of this study have specific auxiliary reference value for clinicians to evaluate the progression of cervical cancer.

This study retrospectively analysed the characteristics of high-risk HPV infection, genotype distribution and cervical cytology in 40,693 women in Putian, but this study has some shortcomings. First, the data were derived from the screening results in the past 2 years, and long-term dynamic observations are lacking. Moreover, considering the limited sample size, the results might not accurately reflect the epidemiological characteristics of HPV in the whole region. Therefore, in a follow-up study, the sample size will be expanded, and the monitoring time will be extended. Second, the HPV detection kit could detect 14 high-risk genotypes. Although it contained the standard typing of the Asian population, not enough types of typing were used, and a risk of missed detection was involved. Third, the women included in this study were aged 30–70. However, a few investigations have focused on HPV infection in people under 30 years old, especially those under 20 years old, which is not conducive to the epidemiological study of HPV in the whole female population.

## Conclusion

In summary, regional HPV screening was conducted to provide valuable clinical guidance for cervical cancer prevention and vaccine selection in this region. Vaccination can effectively prevent HPV infection. It is also an effective first-class measure [22, 46]. Bivalent, tetravalent and nine-valent vaccines are available in the market, and different kinds of HPV vaccines target different HPV genotypes. The results of the present study show that women in Putian could choose a wide range of nine-valent vaccines when vaccinated with the HPV vaccine, which could prevent infection effectively. In addition, HPV infection tends to occur in younger patients. Health departments and medical institutions need to increase scientific publicity about HPV to make women aware of HPV and avoid infection immediately. At the same time, regular and effective screening of HPV-positive middle-aged and older women needs to be focused on to reduce cancer risk.

## Data Availability

The datasets used or analysed during the current study are available from the corresponding author on reasonable request.

## Abbreviations

HPV: Human papillomavirus
CI: Confidence interval
TCT: ThinPrep cytological test
NILM: Negative for intraepithelial lesion and malignancy
ASC-US: Atypical squamous cells of undetermined significance
LSIL: Low-grade squamous intraepithelial lesion
ASC-H: Atypical squamous cells—cannot exclude high-grade squamous intraepithelial lesion
HSIL: High-grade squamous intraepithelial lesion
CIN: Cervical intraepithelial neoplasia
CC: Cervical cancer
